# Digitizing the Appearance of 3D Printing Materials Using a Spectrophotometer

**DOI:** 10.3390/s24217025

**Published:** 2024-10-31

**Authors:** Alina Pranovich, Morten Rieger Hannemose, Janus Nørtoft Jensen, Duc Minh Tran, Henrik Aanæs, Sasan Gooran, Daniel Nyström, Jeppe Revall Frisvad

**Affiliations:** 1 Department of Applied Mathematics and Computer Science, Technical University of Denmark, 2800 Kongens Lyngby, Denmark; apra@dtu.dk (A.P.);; 2 Department of Science and Technology, Linköping University, 602 33 Norrköping, Swedendaniel.nystrom@liu.se (D.N.); 3 3Shape, 1059 Copenhagen, Denmark

**Keywords:** additive manufacturing, appearance modeling, digital twin, model validation, spectral optical properties, radiative transfer, soft proofing

## Abstract

The conventional approach to appearance prediction for 3D printed parts is to print a thin slab of material and measure its reflectance or transmittance with a spectrophotometer. Reflectance works for opaque printing materials. Transmittance works for transparent printing materials. However, the conventional approach does not work convincingly for translucent materials. For these, we need to separate scattering and absorption. We suggest printing a collection of thin slabs of different thicknesses and using these in a spectrophotometer to obtain the scattering and absorption properties of the material. A model is fitted to the measured data in order to estimate the scattering and absorption properties. To this end, we compare the use of Monte Carlo light transport simulation and the use of an analytic model that we developed from the theory of radiative transfer in plane-parallel media. We assess the predictive capabilities of our method through a multispectral photo-render comparison based on the estimated optical properties.

## 1. Introduction

A 3D printed object is created with a descriptive digital twin. This twin is a digital geometric model that sometimes includes material selections for different parts of the object. A sliced version of the digital twin is used as input for the 3D printer when the object is printed. While a conventional digital twin may provide information about material selection, it does not provide sufficient information for the photorealistic rendering of the appearance of the printed object. Optical properties of the different print materials are required to achieve this. Even if the descriptive capability of a digital twin has been updated to include estimates of the optical properties of the print materials, a method is currently missing for the quantitative validation of the digital twin’s ability to describe the appearance of the printed object in a physically based rendering. Our objectives were to provide a practical method for estimating the optical properties of 3D print materials and to suggest a method that enables quantitative photo-render comparisons for testing the appearance prediction capabilities of the estimated optical properties. The overall aim is to make people use photo-render comparisons for the quantitative validation of their digital twin’s ability to describe the appearance of the physical twin.

Translucency is common in 3D printing materials and is apparent when observing different thicknesses of a material within a printed geometry. Appearance evaluation for 3D printing, however, rarely fully captures the translucency of the printing materials [1,2]. The accurate rendering of 3D objects made of translucent materials requires the evaluation of surface and subsurface scattering as well as transmission through the volume. For known optical properties (refractive index, absorption, scattering, roughness), physically based rendering is expected to compute accurate visualizations of such objects. As these parameters cannot be measured directly, common approaches for acquiring optical properties are inverse rendering methods or other methods based on the modeling of the light transport from a well-characterized source illuminating a sample of the studied material [3]. If the detector is a color camera with red, green, and blue (RGB) channels, the obtained optical properties will also be RGB. This is a reduction of the visible spectrum of wavelengths that was designed to describe the trichromatic human visual system. While this perceptually weighted simplification of a spectrum can be sufficient in some applications, it cannot accurately describe effects such as dispersion where light of different wavelengths takes different paths through the material. Thus, as in existing work [4], we set up our acquisition technique to be spectral in order to provide a complete description of the material.

An important but often overlooked step is to quantitatively validate the accuracy of the acquired optical properties when a 3D object is printed using the material of interest. We present a method for the quantitative assessment of the ability to predict the appearance of 3D printed objects using acquired optical properties. To estimate the spectral optical properties of the print materials, we propose an analytic radiative transfer model. Our validation method is useful with other methods for estimating optical properties as well. To demonstrate this, we also tested it with the method by Iser et al. [4]. To the best of our knowledge, we provide the first method for the multispectral validation of optical properties estimated for 3D printing materials through quantitative photo-render comparisons.

To construct a practical model for estimating optical properties, we used the analytic single-scattering solution by Chandrasekhar [5] as an outset. Others have used this model in the past with no refractive interface [6] or with a specular interface [7]. Since 3D printed objects tend to have a rather rough surface, we extended the classic single-scattering model to a version integrated according to a diffuse interface assumption. We performed a series of spectrophotometric measurements using thin plane-parallel samples of different thicknesses. By fitting our model to our observations, we obtained absorption and scattering coefficients of a translucent printing material. The samples needed for this estimation of optical properties are easily 3D printed for any given printing material by simply stacking layers. However, since our model is based on single scattering, it works best for 3D printers that can print with thin layers and use materials of low- to mid-range scattering.

**Figure 1:**
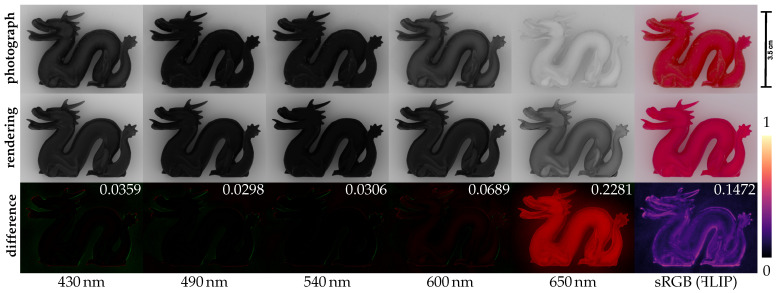
Using thin 3D printed slabs of different thicknesses, we can estimate the spectral optical properties of 3D printing materials. After this estimation, the photorealism of 3D renderings based on these optical properties is, however, unknown. We suggest validating the correctness of acquired optical properties by comparing a multispectral photograph of a 3D printed object with corresponding renderings. In this example, we show differences (green for positive, red for negative) per wavelength and a FLIP error map [8] (values in the colourbar) for the reconstructed RGB image. The white numbers are the root mean squared error for the spectral bands and average FLIP error for sRGB.

Regardless of the estimation method employed, we propose validating the method by testing whether the estimated optical properties can produce a photorealistic rendering of a 3D printed object. To this end, we suggest printing a 3D object and subjecting the object to multispectral imaging in a diffuse lighting environment. Since an expected 3D geometry of the object is available (input for the printer), we can use this to obtain a digital scene aligned with the simple physical setup. This enables us to render images aligned with the captured multispectral images and directly evaluate difference images using a technique such as the FLIP error map [8]; an example is in Figure 1. Such different images are used to quantitatively assess the accuracy of the optical properties. We describe our validation procedure and discuss important aspects to keep in mind when performing this for spectral images, such as when dealing with fluorescence. An advantage of using multispectral images and optical properties is that they directly enable us to assess the visual accuracy for different color spaces using a color difference standard.

Our first contribution is a method to support the use of a conventional spectrophotometer for estimating the spectral scattering properties of a material. Our method requires sheets of different thicknesses with a rough surface. Such sheets are easily obtained from a 3D printer. The benefit of our approach is that we avoid extensive Monte Carlo light transport simulation. Our method is, however, limited to materials that do not exhibit significant multiple scattering when prepared as thin slabs of submillimeter thicknesses. In addition to this, we contribute a procedure for assessing the photorealism of the rendered appearance of parts to be 3D printed using materials with estimated scattering properties.

## 2. Related Work

As advocated by Greenberg et al. [9], an important stage in the validation of realistic image synthesis is to verify the correctness of the input and rendering technique by radiometric comparison after light transport simulation. One way to perform such a radiometric comparison is by comparing photographs with renderings and computing difference images [10,11]. Such a comparison is common during the acquisition of object appearance [12] or optical properties [13], or both [14]. When a 3D printer is available, we can acquire optical properties in a more straightforward way using simpler printable geometries [4,15]. We can even obtain spectral properties in this way [4]. The simple geometries used for the acquisition of the optical properties are then not suitable 3D objects for the radiometric validation. We propose printing a 3D object and carrying out a photo-render comparison to verify the correctness of acquired spectral optical properties.

Our validation method is independent of the choice of 3D printer, printing material(s), method for acquiring optical properties, and rendering technique. The ease of use of the full procedure is, however, a key concern of ours. Printers are easy to use, and rendering is easily performed using an off-the-shelf rendering tool. To ease the acquisition of optical properties, our method is based on an analytic model for the light transport in a thin plane-parallel sample. Such thin slabs of varying thicknesses are easily printed, and analytic models for the light transport in a thin slab are commonly available [5,16,17]. Existing methods [4,13,14,15] rely on computationally expensive Monte Carlo simulation. Since the estimation of optical properties is based on optimization, a loss function involving Monte Carlo simulation makes the process heavy. Our approach is a light-weight alternative.

Iser et al. [4] suggested using Monte Carlo path tracing to precompute appearance maps, which are plots for various optical properties of sample reflectance on black versus white backgrounds. The measurement of reflectance on black and white backgrounds can be inserted in these plots, and the interpolation of simulated nearest neighbors leads to an estimate of the optical properties. Our analytic model avoids the extensive precomputation of appearance maps that they suggest and eases the use of an optimization loop to estimate the optical properties. The disadvantage of our analytic model is that it is based on a single-scattering model. If a model is needed that includes multiple scattering, we suggest using the Monte Carlo precomputation-based method by Iser et al. [4].

Analytic models have been used extensively in the past for acquiring optical properties [3]. In the case of highly scattering materials, Jensen et al. [18] used a dipole solution based on diffusion theory and assumed a half-space sample. This approach has been extended to heterogeneous samples [19,20,21] and heterogeneous slabs [22]. A diffusion-based method is even available for estimating optical properties based on a single image captured in the wild [23]. The assumption of a highly scattering material, however, makes diffusion-based models unsuitable for optically thin materials. For liquid materials, Narasimhan et al. [24] used dilution to end up in the single-scattering regime, where an analytic model is more easily obtained. In our case, we can print thin slab samples that bring us to the single-scattering regime. In addition, we can print a 3D object made of the same material(s) and perform radiometric validation.

In assuming a smooth interface between layers, analytic thin slab models have been used for estimating the optical properties of surface contaminants [25]. In the context of a rough surface, the single-scattering microfacet model by Walter et al. [26] is often employed, and Dai et al. [27] proposed a model for estimating spatially varying roughnesses of the top and bottom surfaces of a thin slab with no volume scattering. While we used a multiple-scattering version of the microfacet surface scattering model [28] for rendering during the validation, we assumed a diffusely scattering surface in our thin slab model for the acquisition of optical properties. This is to keep the model analytic and practical. To estimate the optical properties of paper, Papas et al. [29] presented a thin-slab model combining surface and subsurface scattering. This is similar to ours but the complexity of their model made them precompute a tabulated version for the fitting of the model to observations. Papas et al. [29] listed the verification of the physical accuracy of the acquired optical properties as future work. Our validation procedure is suitable for this and other acquisition approaches that apply to sample geometries that we can 3D print (including the approach of Iser et al. [4], as we demonstrate in our results).

Chen and Urban [30,31] proposed a different method of material photometric characterization and introduced a deep learning approach for color prediction. Instead, we propose the physically based modeling of the spectrophotometric measurements of the reflectance and transmittance of translucent materials. We use directly 3D printed patches of different thicknesses for the measurements. Such samples are easily obtained with most 3D printing techniques and will have a somewhat rough surface due to the printing process. A model simpler than ours could likely be derived for a smooth surface, but this would require additional polishing effort. The number of different sample thicknesses needed for our method mainly depends on the width of the interval in which color variation is visible when the samples are placed on a black background.

## 3. Instruments and Materials

To estimate the spectral properties of a printing material, we used thin slabs of different thicknesses. We printed samples with thicknesses of 0.1 to 1.0 mm, with steps of 0.05 to 0.1 mm using Stratasys Vero photopolymer 3D printing materials. Our samples were printed on a J850 (Stratasys, Rehovot, Israel), which is a PolyJet 3D printer. The printed samples had rough surfaces that we left without post-processing.

**Figure 2:**
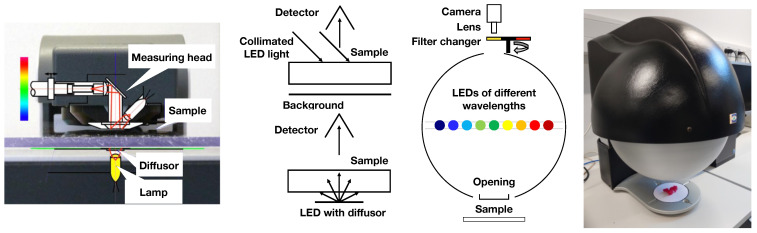
**Left**: Spectrophotometer measurement of diffuse transmission and reflection. For the transmission measurement, we have a diffuse light source with a circular cross section (9 mm diameter). For the reflection mode, the sample is illuminated by a collimated light source with a circular cross-section and 9 mm diameter (at an angle of incidence of 45°). The detection aperture is in both cases an 8 mm wide circle with an acceptance angle (0 ± 2)°. **Right**: VideometerLab instrument for the multispectral imaging of a sample that is diffusely illuminated due to the use of an integrating sphere. The sketch of this instrument is courtesy of Videometer, www.videometer.com (accessed on 27 October 2024).

For the acquisition of optical properties, we used a spectrophotometer developed for color management in conventional printing on paper. Specifically, we used a Spectro LFP S2 (Barbieri Electronic, Bressanone, Italy). The configuration of the instrument is illustrated in Figure 2. In reflectance mode, the sample is illuminated by three collimated LED beams, each directed at 45° to the surface normal. In transmission mode, the sample is backlit by a diffuse source with a circular aperture of 9 mm in diameter. Reflected and transmitted light is detected at 0° (the normal direction). In reflectance mode, we used black and white backgrounds. Reflected and transmitted spectral power distributions were measured within the visible part of the spectrum: 380 to 780 nm with 10 nm steps.

For validation, a 3D object is printed using the material of interest. We then acquire images using a multispectral imaging device with an integrating sphere. Specifically, we used a VideometerLab 4 (Videometer, Herlev, Denmark). In this device, grayscale images of a scene are collected under diffuse illumination at individual narrow wavebands in the range between 365 and 970 nm. Obtained grayscale values then correspond to the spectral radiance computed in a spectral rendering. We modeled the camera in VideometerLab as a pinhole camera, with two radial distortion coefficients. The intrinsic parameters and normal of the background plane were obtained by camera calibration with a checkerboard.

## 4. Spectrophotometer Model for Estimating Optical Properties

With an outset in thin slab samples and the spectrophotometer configuration, we built a plane-parallel light transport model describing reflectance under collimated illumination and transmittance under diffuse illumination. This is useful for our setup with collimated light incident at an oblique angle for reflectance measurement and a diffuse source underneath the sample for transmittance measurements. We assumed a perfectly rough surface exhibiting Lambertian reflection and transmission.

We modeled the detector of an instrument as a pinhole camera with pinhole position p, so that the observed radiant flux $\Phi_{o}$ is only due to radiance reflected or transmitted toward p from the observed area $A_{o}$. We let $f_{s}$ denote the bidirectional scattering distribution function (BSDF) of a sample, while $\omega_{i}$ and $\omega_{o}$ are the solid angles of the incident and observed light. Assuming uniform illumination of the sample by an irradiance *E*, we set up a measurement equation for the instrument:

(1) $$\begin{array}{ccc} \rho & = & \frac{1}{\rho_{\mathrm{ref}}}\frac{\Phi_{o}}{EA_{o}}=\frac{1}{\rho_{\mathrm{ref}}}\frac{\int_{A_{o}}\int_{\omega_{o}}L_{o}\cos\theta_{o}^{\prime }\;d\omega_{o}^{\prime }\;dA_{o}^{\prime }}{EA_{o}}\\ & = & \frac{1}{\rho_{\mathrm{ref}}}\frac{\int_{A_{o}}\int_{\omega_{o}}\int_{\omega_{i}}f_{s}\;dE({\vec{\omega }}_{i})\cos\theta_{o}^{\prime }\;d\omega_{o}^{\prime }\;dA_{o}^{\prime }}{EA_{o}}\;,\; \end{array}$$

where ρ is the measured reflectance value, and $\rho_{\mathrm{ref}}$ is normalization by a reference measurement captured during calibration of the instrument. Another way to write the innermost integral of the measurement equation is

(2) $$\int_{\omega_{i}}f_{s}\left(x,{\vec{\omega }}_{i},{\vec{\omega }}_{o}\right)\;dE=\int_{4\pi }f_{s}\left(x,{\vec{\omega }}_{i},{\vec{\omega }}_{o}\right)L_{i}\left(x,{\vec{\omega }}_{i}\right)\left|\cos\theta_{i}\right|\;d\omega_{i}\;,$$

where $L_{i}$ is incident radiance, and $\theta_{i}$ is the angle of incidence. To model the outcome of a measurement with this instrument, we need expressions for $f_{s}$ and $\rho_{\mathrm{ref}}$.

By definition, a BSDF is the ratio of observed radiance $L_{o}\left(x,{\vec{\omega }}_{o}\right)$ at a (macroscopic) surface location x in the direction ${\vec{\omega }}_{o}$ to the irradiance $E(x,{\vec{\omega }}_{i})$ incident from a direction ${\vec{\omega }}_{i}$:

(3) $$f_{s}=\frac{dL_{o}}{dE}\;.$$

In reflection mode, we measure normalized reflectance ρ by illuminating a sample at an angle $\theta_{e}$ using a collimated source. The incident radiance is then

(4) $$L_{i}\left(x_{i},{\vec{\omega }}_{i}\right)=E\left(x_{i}\right)\;\frac{\delta ({\vec{\omega }}_{i}-{\vec{\omega }}_{e})}{|\cos\theta_{i}|}\;,$$

where *E* is the irradiance at $x_{i}$ due to the source, and ${\vec{\omega }}_{e}$ is the direction toward the source. We let $A_{i}$ refer to the illuminated area. The source illuminates the sample uniformly in $A_{o}$ with $A_{i}>A_{o}$.

With $\theta_{e}$ constant, we can reasonably assume that the white reference material acts like a perfect diffuser with $\rho_{\mathrm{ref}}=1/\pi$, and the BSDF becomes a bidirectional reflectance distribution function (BRDF) $f_{s}=f_{r}$. The reference measurement in the transmission mode directly observes the source, which is assumed to be perfectly diffuse so that $\rho_{\mathrm{ref}}=1/\pi$ and the BSDF is a bidirectional transmittance distribution function (BTDF): $f_{s}=f_{t}$. Our material sample is a thin layer with a surface BSDF for each of the top and bottom surfaces and also scattering and absorption within the volume. If the plane-parallel volume is optically thin, we can use a single scattering solution [5] (see §63) as an expression for the BSDF of the volume.

Using Equations (2) and (4), we have

(5) $$\int_{\omega_{i}}f_{r}\left(x,{\vec{\omega }}_{i},{\vec{\omega }}_{o}\right)\;dE=f_{r}\left(x,{\vec{\omega }}_{e},{\vec{\omega }}_{o}\right)\;E\;.$$

If we assume that the collection cone of the detector is small enough to justify $\theta_{o}=0^{\circ }$ everywhere, the measurement Equation (1) then becomes

(6) $$\rho_{r}=\pi f_{r}\left({\vec{\omega }}_{e},\vec{n}\right)\;,$$

where the *r* subscript signals reflection mode, and $\vec{n}$ is the direction of the sample surface normal. Thus, given an analytic expression for the BRDF, we have a model of the measurement.

In transmission mode, we have diffuse incident illumination with $L_{i}=E/\pi$ and

(7) $$\int_{\omega_{i}}f_{t}\left(x,{\vec{\omega }}_{i},{\vec{\omega }}_{o}\right)\;dE=\int_{-2\pi }f_{t}\left(x,{\vec{\omega }}_{i},{\vec{\omega }}_{o}\right)\left|\cos\theta_{i}\right|\;d\omega_{i}\;\frac{E}{\pi }\;.$$

The measurement equation is then

(8) $$\rho_{t}=\int_{-2\pi }f_{t}\left({\vec{\omega }}_{i},\vec{n}\right)\left|\cos\theta_{i}\right|\;d\omega_{i}=\rho_{td}+\rho_{ts}\;,$$

where, after the second equality, we split the integral into one term for direct transmission $\rho_{td}$ and another for scattering within the layer $\rho_{ts}$. With our assumption of a diffuse surface BSDF and in expressing direct transmission using the law of exponential attenuation, this part of the observed light is

(9) $$\rho_{td}=\frac{{\left(1-\rho_{d}\right)}^{2}}{\pi }\int_{-2\pi }e^{-\frac{\sigma_{t}t}{\cos\theta_{t}}}\cos\theta_{t}\;d\omega_{t}\;,$$

where $\rho_{d}$ is the diffuse reflectance of the top and bottom surfaces of the layer, $\sigma_{t}$ is the extinction coefficient of the sample material, *t* is the sample layer thickness, and $\theta_{t}$ is the angle of transmission of the light through the layer. Chandrasekhar [5] (see §60,§92) describes integrals of the form

(10) $$E_{n}\left(x\right)=\int_{0}^{1}e^{-\frac{x}{\mu }}\mu^{n-2}\;d\mu \;\left(x>0\right)\;.$$

The integral we need for $\rho_{td}$ is then $2\pi E_{3}$.

We can separate the BRDF of a uniformly illuminated translucent layer into insurface and subsurface scattering effects [32]. The part of the light that is not reflected from the surface is transmitted into the material and may undergo scattering and absorption according to radiative transfer theory [5]. Light is further transmitted to the bottom surface of the material sample and may be scattered back or transmitted through to be scattered back or absorbed by a background material. The part of the subsurface scattered light reemerging from the scattering medium will contribute to the macroscopic BRDF of the material. Suppose the surface is a perfect diffuser; then,

(11) $$f_{r}=\frac{\rho_{d}}{\pi }+\frac{{\left(1-\rho_{d}\right)}^{2}}{\pi^{2}}\int_{+2\pi }\int_{-2\pi }f_{rs}\left({\vec{\omega }}_{t},{\vec{\omega }}_{s}\right)\cos\theta_{t}d\omega_{t}\cos\theta_{s}d\omega_{s}\;,$$

where $f_{rs}$ is the BSDF describing the ratio of radiance scattered back to the surface from the material sample (or something beneath it) due to an element of irradiance transmitted through the top surface of the material sample in the direction ${\vec{\omega }}_{t}$. With no dependency on the azimuthal angle, the relation between solid angles and spherical coordinates is useful for simplifying the integrals in Equation (11). Using the notation where μ is the cosine of the inclination angle (e.g., $\mu_{s}=\cos\theta_{s}$), we have

(12) $$f_{r}=\frac{\rho_{d}}{\pi }+4{\left(1-\rho_{d}\right)}^{2}\int_{0}^{1}\int_{0}^{1}f_{rs}\left(\mu_{t},\mu_{s}\right)\;\mu_{t}d\mu_{t}\;\mu_{s}d\mu_{s}\;.$$

The next step is to separate $f_{rs}$ into bottom surface $f_{bs}$, background $f_{bg}$, and volume scattering effects $f_{ss}^{+}$:

(13) $$f_{rs}=f_{bs}+f_{bg}+f_{ss}^{+}\;.$$

Considering both top and bottom surfaces as perfect diffusers, we have

(14) $$f_{bs}=\frac{\rho_{d}}{\pi }e^{-\sigma_{t}t\left(\frac{1}{\mu_{t}}+\frac{1}{\mu_{s}}\right)},$$

and one way to describe $f_{ss}^{+}$ is using a model for single scattering in a layer. In such a model, the reflection (backscattering) from a translucent layer would be [5,6]

(15) $$f_{ss}^{+}\left(\mu_{t},\mu_{s}\right)=\Lambda \;p\left(\mu_{t},\mu_{s}\right)\frac{\mu_{t}}{\mu_{s}+\mu_{t}}\left(1-e^{-\tau \left(\frac{1}{\mu_{t}}+\frac{1}{\mu_{s}}\right)}\right),$$

where Λ∈[0,1) is the scattering albedo; *p* is the phase function, which describes the directional distribution of the scattered light; and $\tau =\sigma_{t}t$ is the optical thickness of the layer. Assuming that the material exhibits isotropic scattering, we have $p=\frac{1}{4\pi }$.

The integrals in Equation (12) for $f_{bs}$ or $f_{ss}^{+}$ can be solved analytically using exponential integrals $E_{n}$ and $F_{n}$ available from Chandrasekhar [5]. We need $E_{3}$ for $f_{bs}$ and $F_{3}$ for $f_{ss}^{+}$. The numerical evaluation of $E_{1}$ is available (see below). Recurrence relations for $E_{n}$ and $F_{n}$ are [5]

(16) $$\begin{array}{ccc} nE_{n+1}\left(x\right) & = & e^{-x}-xE_{n}\left(x\right)\;\left(n\geq 1\right)\;, \end{array}$$

(17) $$\begin{array}{ccc} F_{j}\left(\tau ,\mu \right) & = & \mu \left(F_{j-1}\left(\tau ,\mu \right)+e^{\frac{\tau }{\mu }}E_{j}\left(\tau \right)-\frac{1}{j-1}\right). \end{array}$$

Using Equation (16), we find

(18) $$E_{3}\left(x\right)=\frac{1}{2}(e^{-x}\left(1-x\right)+x^{2}E_{1}\left(x\right)).$$

Given $F_{1}$ and $F_{2}$, we can then find $F_{3}$ using Equation (17). The following solution is available for $F_{1}$ when the second argument is negative:

(19) $$\begin{array}{c} F_{1}\left(\tau ,-\mu \right)=\mu \left[\ln\left(1+\frac{1}{\mu }\right)-e^{-\frac{\tau }{\mu }}E_{1}\left(\tau \right)+E_{1}\left(\tau +\frac{\tau }{\mu }\right)\right]. \end{array}$$

Using the recurrence relation for $E_{n}$ (Equation (16)), we have

(20) $$F_{2}\left(\tau ,-\mu \right)=\mu (1-F_{1}\left(\tau ,-\mu \right)+e^{-\frac{\tau }{\mu }}\left(\tau E_{1}\left(\tau \right)-e^{-\tau }\right)),$$

which means that we can directly evaluate $E_{3}$ and $F_{3}$ if we just have an implementation of $E_{1}$ (called expint in Matlab and exp1 in Python SciPy). Alternatively, these integrals can be efficiently represented by a linear combination of two exponential functions of the material’s optical thickness:

(21) $$G_{i}\left(\tau \right)=a_{i}\;e^{b_{i}\;\tau }+c_{i}\;e^{d_{i}\;\tau },$$

where $G_{i}$ is either $E_{3}$ or an integral over $F_{3}$, and $a_{i},b_{i},c_{i},d_{i}$ are fitted to numerical evaluations of the mentioned integrals.

For a black background ($f_{bg}=0$), and assuming isotropic scattering, we have

(22) $$\rho_{r}=\rho_{d}+{\left(1-\rho_{d}\right)}^{2}\left(4\rho_{d}E_{3}^{2}\left(\tau \right)+\Lambda \int_{0}^{1}\;\;F_{3}\left(\tau ,-\mu_{s}\right)\;d\mu_{s}\right),$$

where we, for efficiency, can replace $\int_{0}^{1}\;\;F_{3}\left(\tau ,-\mu_{s}\right)\;d\mu_{s}$ and $E_{3}\left(\tau \right)$ with $G_{i}$ functions and use those when fitting.

Returning to the transmission measurement, we can use the same single-scattering model to find

(23) $$\rho_{ts}=\frac{{\left(1-\rho_{d}\right)}^{2}}{\pi }4\pi^{2}\;\int_{0}^{1}\;\;\int_{0}^{1}\;\;f_{ss}^{-}\left(\mu_{t},\mu_{s}\right)\mu_{t}d\mu_{t}\mu_{s}d\mu_{s}\;.$$

with [5,6]

(24) $$f_{ss}^{-}\left(\mu_{t},\mu_{s}\right)=\Lambda \;p\left(\mu_{t},\mu_{s}\right)\frac{\mu_{t}}{\mu_{s}-\mu_{t}}\left(e^{-\frac{\tau }{\mu_{s}}}-e^{-\frac{\tau }{\mu_{t}}}\right).$$

To obtain an expression for the integrand of the outer integral in Equation (23), we again use the $F_{3}$ integral. In transmission mode, Equation (1) for isotropic scattering then becomes

(25) $$\rho_{t}={\left(1-\rho_{d}\right)}^{2}\left(2E_{3}\left(\tau \right)+\pi \Lambda \int_{0}^{1}\;\;e^{-\frac{\tau }{\mu_{s}}}F_{3}\left(\tau ,\mu_{s}\right)\;d\mu_{s}\right).\;$$

In the case of a Lambertian background with $f_{bg}\neq 0$, we utilize our previous results of $\rho_{t}$ and combine them with $\rho_{r}$ such that

(26) $$\rho_{r}^{bg}=\rho_{r}+\rho_{t}^{2}\;\rho_{bg}\;,$$

where $\rho_{bg}$ is a diffuse reflectance value measured separately.

The validity of the exact analytical expressions approximating the single-scattering contribution is limited to low optical thicknesses. The obtained measured values of the reflectance of the samples can suggest whether or not the contribution of the multiple subsurface scattering is negligible. For a given single-scattering albedo, the reflectance due to full multiple scattering can be approximated through the multiplication of the terms involving $F_{3}$ with the ratio of multiple to first-order scattering [17]. We did not use a factor based on this ratio to model multiple scattering in our experiments.

### Varying Translucency and Material Mixtures

**Figure 3:**
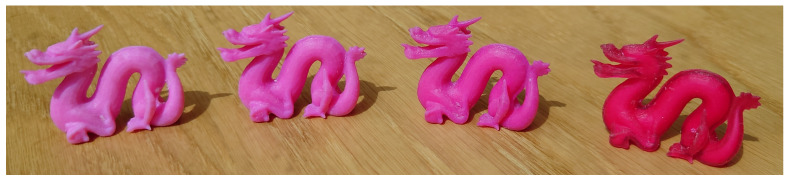
PolyJet 3D printed objects with different material mixtures of varying translucency (blending the red primary with white material).

PolyJet 3D printing allows the blending of two different materials within the volume. This is useful for achieving different colors, by mixing primaries, and varying levels of translucency, by mixing the primary with the white material. The optical properties of the mixture can be estimated with the weighted average according to the material proportions:

(27) $$\begin{array}{cc} \sigma_{a}\left(w_{1},w_{2}\right) & =w_{1}\;\sigma_{a1}+w_{2}\;\sigma_{a2}\\ \sigma_{s}\left(w_{1},w_{2}\right) & =w_{1}\;\sigma_{s1}+w_{2}\;\sigma_{s2}, \end{array}$$

where $w_{1}$ and $w_{2}$ are fractional presences of each material. In our experiments, we used a combination of the red primary with white in ratios of 0.7 and 0.3 or 0.5 and 0.5 to have the different material mixtures seen in Figure 3.

## 5. Radiometric Validation Procedure

**Figure 4:**
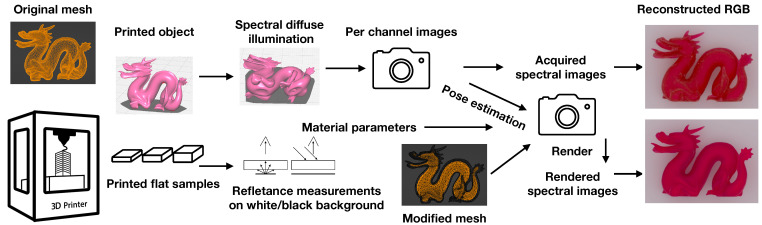
Schematic of our radiometric validation procedure. Note that the printed object exists both physically and digitally and the pose estimation of the digital twin is based on the captured multispectral image of the physical object and calibration of the camera.

An overview of our validation procedure is in Figure 4. Our approach relies on 3D printing and requires a method for the spectral acquisition of the optical properties of the printing material of interest. The method presented in Section 4 is an option. The optical properties could also be computed based on a material characterization [33,34] or through the adjustment of reference properties [35]. Once optical properties are available, our procedure consists of the following steps:

1. Print a 3D object using the material of interest. Select an object, preferably with regions of different material thicknesses. The triangle mesh describing the object will be used both for printing and for creating the digital twin of the printed object that will be used for the photo-render comparison. We used a lowpoly version of the Stanford dragon [36,37] prepared for 3D printing (a non-trivial 3D geometry with both thin and thick regions).
2. Adjust the digital twin of the object according to the printing process. A physically printed object is modified by the printing process. For our validation procedure, we need a digital twin that resembles the physical object as much as possible. In the case of 3D printing by fused filament fabrication, one can convert the tool path computed during slicing into a triangle mesh to be used as the digital twin of the object. The resulting mesh consists of tubes following the tool path and will thus exhibit the well-known staircasing artifacts seen in such printed objects. We used a PolyJet printer and the internal volume was filled with a solid infill material. Thus, we converted the mesh to a signed distance field, picked an appropriate negative value, and polygonized the internal isosurface [38] to create a triangle mesh interface between the material of interest and the infill material.
3. Set up a diffuse lighting environment with a multispectral source and/or camera. To capture reference images of the printed object, we opted for a diffuse lighting environment, as this is easily modeled in rendering, with a plane for the object to lie on and a constant background radiance. Multispectral images should be captured using different narrowband sources and/or filters for the camera. The physical object was placed on a flat uniformly colored matte surface (we used paper), and the digital twin needed to be the same. We used Blender’s rigid body physics [39] to let the digital object fall naturally on a plane. The physical camera was placed to observe the object, and a pose estimation of the object was needed to place the virtual camera accordingly.
4. Align reference photos with a rendering of the digital twin. Through camera calibration, we obtained a camera matrix and the normal of the plane that the object was lying on. From the camera matrix, we computed the intrinsic parameters needed for rendering images taken with the camera (focal length, sensor width, principal point). To estimate the pose of the object relative to the camera, we used a variation of the method by Hannemose et al. [40]. Their method assumes a point light source and six degrees of freedom for the pose. Since our physics simulation estimates how the object is lying on the plane underneath, we only needed to estimate the rotation around the normal and the (x,y,z) position of the object. We used a manually segmented silhouette of the object that we aligned with the rendered silhouette. Since our lighting was diffuse, we did not need to estimate the position of the light source. For our digital scene with the printed object lying on a plane illuminated by a constant background, the alignment provided us with extrinsic and intrinsic camera parameters that we directly used for rendering images aligned with the reference photographs.
5. Compare photo-renders using the estimated optical properties. The diffuse reflectance of the plane and the constant radiance of the background were selected to best match the reference images. For the PolyJet infill material, we used the optical properties estimated for Vero Pure White by Elek et al. [15]. These become input for volume path tracing [41] together with the optical properties estimated for the material of interest in the validation procedure. We could then render grayscale images for individual wavebands and assess the accuracy of the properties by inspecting difference images (or FLIP error maps) and using full-image perceptual error metrics (such as the average FLIP error). For an overall appearance comparison, we used the spectral images to reconstruct an RGB image (as exemplified in Figure 1).

## 6. Implementation

For 3D printed thin slabs of varying thicknesses, for each wavelength λ, we used our model (Section 4) to plot a curve describing the expected reflectance $\rho_{r}^{bg}\left(t\right)$ or transmittance $\rho_{t}\left(t\right)$ of the sample as a function of its thickness *t*. The parameters needed as input for the model are the scattering albedo Λ, the extinction coefficient $\sigma_{t}$, the diffuse surface reflectance $\rho_{d}$, and the background reflectance $\rho_{bg}$. A spectrophotometer was used to measure the spectral curves of the reflectance $\rho_{r}^{bg}\left(\lambda \right)$ for a black and a white background as well as transmittance $\rho_{t}\left(\lambda \right)$. Performing a set of spectrophotometer measurements for each sample, we obtained measured reflectances and transmittances for different thicknesses that we plotted as reference points and fitted our model to. The fit provides an estimate of the input parameters (Λ, $\sigma_{t}$, $\rho_{d}$).

We set up a nonlinear least squares optimizer to fit the curves described by Equations (22), (25) and (26) to the obtained reflectance and transmittance data. For the background reflectances, we used measured values of backgrounds instead of assuming them to be 1 (white) and 0 (black). We set up the fitting procedure in two steps. As the first step, the surface reflectance parameter $\rho_{d}$ was fitted (constant across the spectrum) by minimizing the sum of the residuals when fitting the absorption and scattering coefficient for five wavelengths (430, 500, 550, 580, and 650 nm). With $\rho_{d}$ fixed, we fitted the absorption and scattering coefficients, $\sigma_{a}$ and $\sigma_{s}$, respectively, for the whole spectrum, each wavelength independently. This was achieved using $\tau =\sigma_{t}t$ for the optical thickness, where *t* is the sample thickness, $\sigma_{t}=\sigma_{a}+\sigma_{s}$ for the extinction coefficient, and $\Lambda =\sigma_{s}/\sigma_{t}$ for the scattering albedo. As mentioned in Section 4, a wavelength-dependent correction factor can be introduced in our model and fitting to help the model fit reflectance and transmittance using samples exhibiting multiple scattering. We did not use this correction in the fits reported in Section 7.

For our radiometric validation procedure, we set up a digital scene in Blender using camera parameters from the camera calibration and the pose estimation. We imitated diffuse lighting conditions by setting the world’s background illumination to a constant. For shading, we used unidirectional volume path tracing [41] with the estimated absorption and scattering coefficients as input. For surface scattering, we needed to select a roughness parameter. This is not the same as $\rho_{d}$, but we used $\rho_{d}$ as inspiration for selecting the surface roughness, as the parameters are related. For efficient rendering, we used a custom-built renderer implemented in the NVIDIA OptiX framework [42]. Another renderer supporting the path tracing of a volume with a rough surface using physically based optical properties could be used instead (e.g., PBRT [41]). Since the printed objects have relatively high surface roughness, we found it important to include multiple insurface scattering when a light path interacts with the surface of a volume. This was needed to convincingly render the appearance of the objects. We implemented multiple insurface scattering using a method similar to the one described by Wang et al. [43].

**Figure 5:**
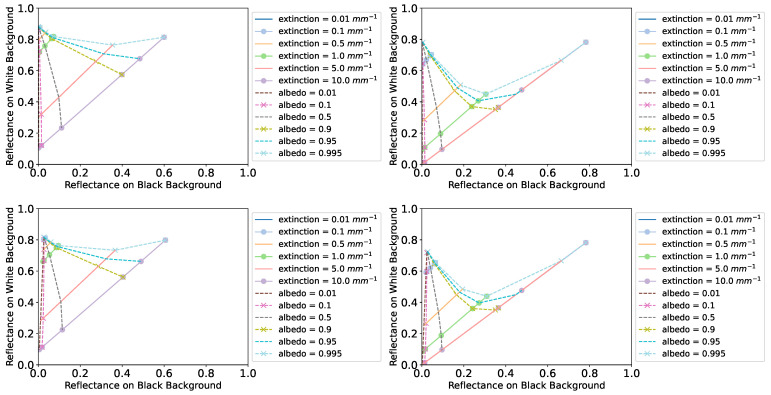
Appearance maps generated with Monte Carlo path tracing simulations for layer thicknesses of 0.1 mm (**left** column) and 1.0 mm (**right** column). The surface roughness was set to 0.03, and the reflectances of the white and black backgrounds were set to 1 and 0, respectively, in the **top** row but 0.93 and 0.03 in the **bottom** row.

To test our validation procedure with a different method for estimating the optical properties of 3D printing materials, we also followed the procedure by Iser et al. [4] and estimated optical properties using Monte Carlo-simulated appearance maps. An appearance map is a one-to-one mapping in which

$$\left(\Lambda ,\sigma_{t}\right)\mapsto \left(\rho_{r}^{\mathrm{black}},\rho_{r}^{\mathrm{white}}\right)\;,$$

where $\rho_{r}^{\mathrm{black}}$ and $\rho_{r}^{\mathrm{white}}$ are $\rho_{r}^{bg}$ for black and white backgrounds, respectively. For a given combination of optical properties, we can run a pair of Monte Carlo path tracing simulations to find $\left(\rho_{r}^{\mathrm{black}},\rho_{r}^{\mathrm{white}}\right)$. The visualization of the appearance map is a plot with $\rho_{r}^{\mathrm{black}}$ along the horizontal axis and $\rho_{r}^{\mathrm{white}}$ along the vertical axis. Each simulated pair is a point in this plot and corresponds to a set of optical properties. Figure 5 shows some appearance map examples. A measured pair of reflectances at wavelength λ for a given sample of thickness *t* is a point in the plot. By finding the nearest simulated neighbor points, one can use linear interpolation between their corresponding $\left(\Lambda ,\sigma_{t}\right)$ pairs to find the optical properties estimated by the appearance map for the given sample and wavelength.

The appearance map interpolation method requires a Monte Carlo path tracing simulator for a light scattering plane-parallel volume with a rough surface. We used a simulator of this kind that was available from previous work [44]. This enabled us to simulate reflectance values on white and black backgrounds for the sample thicknesses used in our experiment using the surface roughness parameter selected to represent $\rho_{d}$. Following the described method, we estimated a set of optical properties by interpolating the simulated values to match the measured ones.

**Figure 6:**
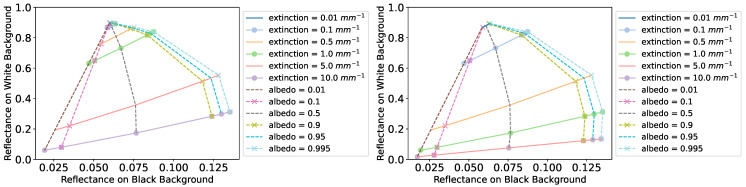
Appearance maps plotted using our model for layer thicknesses of 0.1 mm (**left**) and 1.0 mm (**right**). Roughness was set to 0.016, and the reflectances of the white and black backgrounds were set to 0.93 and 0.03, respectively. This plot illustrates the single-scattering limitation of our model.

A challenge for the method of Iser et al. [4] is that parameter estimation requires the generation of several appearance maps as they change for different sample thicknesses and each point in an appearance map requires a pair of simulations with white and black backgrounds. Other parameters that require recomputing the appearance maps include the background reflectance and surface roughness. Since the appearance maps shift with these parameters, the number of simulations needed quickly grows, making the method quite impractical compared with our analytic model. We decided to use measured background reflectance values at a wavelength of 650 nm. The advantage of using Monte Carlo simulation is that it includes multiple scattering. Our model has continuous functions and is more easily used in an optimizer, but if we try to plot its predicted reflectances on black and white background as an appearance map, the predicted values stop matching the Monte Carlo simulation well when the scattering coefficient increases above $2\;{\mathrm{mm}}^{-1}$ for a sample of 0.1 mm thickness. Figure 6 shows examples of appearance map prediction based on our model. It is clearly limited by the single-scattering assumption.

When using the appearance maps for estimating optical properties, we used a denser set of simulations than the ones plotted to reduce interpolation artifacts. When estimating optical properties using our model, the appearance map only serves the purpose of providing an initial guess for the fitting procedure. The results and comparison of the methods are described in the next section.

## 7. Results

**Figure 7:**
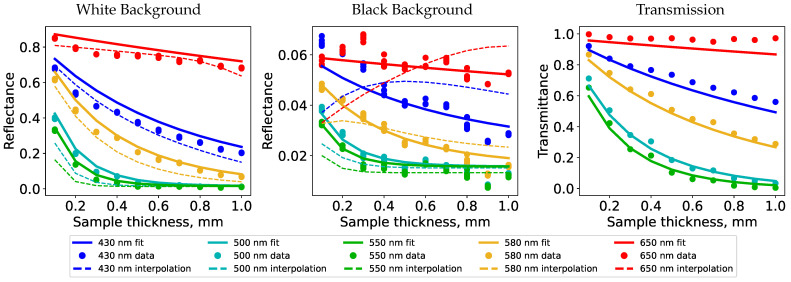
Fit of our model (solid curves) to the spectrophotometer measurements (dots). The dashed curves were generated using Monte Carlo simulation with parameters estimated using appearance map interpolation.

In the first step of our fitting procedure for estimating optical properties, the diffuse reflectance value describing the surface roughness was estimated to be $\rho_{d}=0.016$. With this value fixed, we fitted our thin-slab model to the measured reflectance values of the samples on white and black backgrounds as well as the measured transmittance values. In parallel, we generated reflectance values with Monte Carlo path tracing simulation [44] to create appearance maps and find the optical properties through their interpolation [4]. Background reflectances were measured to be 0.93 for white and 0.03 for black backgrounds at a wavelength of 650 nm. We selected a surface roughness inspired by the surface reflectance obtained from our fitting procedure. The estimated absorption and scattering coefficients slightly varied for each sample thickness. We chose to average the estimated optical properties and compare them to the ones obtained by fitting our analytical model. The measured data and results based on estimated optical properties are compared at selected wavelengths in Figure 7 for the case of Vero Red 3D printing material. These plots include curves generated with the path tracing simulation using the parameters obtained from interpolation based on the appearance maps (dashed lines).

The poor match of the Monte Carlo simulated reflectances with the measured data is due to the use of interpolation between precomputed simulations for a discrete set of selected optical properties and the averaging of different estimated properties for different sample thicknesses (as prescribed by Iser et al. [4]). An optimization would be expected to provide a better match, but this would require an evaluation of the computationally expensive Monte Carlo simulation within the optimization loop. This is not prohibitive; it is just impractical in comparison to using an analytic model.

**Figure 8:**
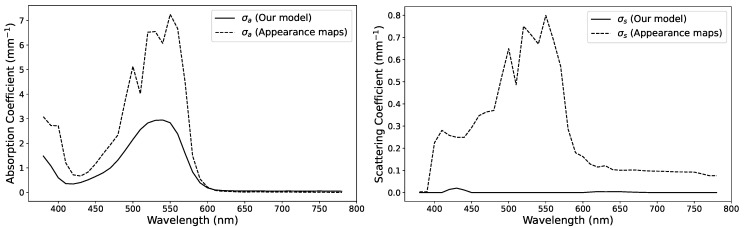
Estimated spectral absorption coefficients ($\sigma_{a}$, **left**) and scattering coefficients ($\sigma_{s}$, **right**) of the Red Vero material when using our model (solid curves) and appearance maps averaged over 10 values for sample thicknesses of 0.1 to 1 mm (dashed curves).

**Figure 9:**
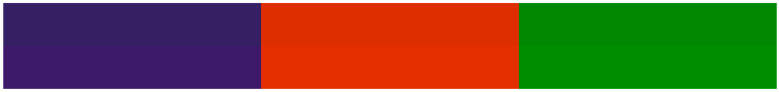
Mixtures of primary materials for samples of 1 mm thickness as measured (top) and reconstructed using our estimated optical properties (bottom). Colors are as observed on a white background. From left to right, 1:1 mixtures of Red and Blue, Red and Yellow, and Yellow and Blue.

The absorption and scattering coefficients that we estimated for Vero Red using the two methods are shown in Figure 8. We first tested our material parameters for the reconstruction of the color of 1 mm samples produced with 1:1 mixtures of each pair of Vero material primaries measured with our spectrophotometer on white background, as shown in Figure 9. After setting up the 3D scene, the spectral images were reconstructed in an sRGB space under D50 illumination. Figure 1 shows a reconstructed RGB image and the spectral photographs that it is based on as well as corresponding renderings from our radiometric validation procedure. The photo-render alignment enabled us to compute difference images as well as a FLIP error map [8], which is an error map that highlights perceptual differences between images. It is clear from the difference images that we overestimated the absorption coefficient at the longer wavelengths. As seen in the compared RGB images, the precise source of such a slight deviation in the predicted appearance from the photographed appearance would not be clear if the validation procedure had not included a stack of spectral images.

**Figure 10:**
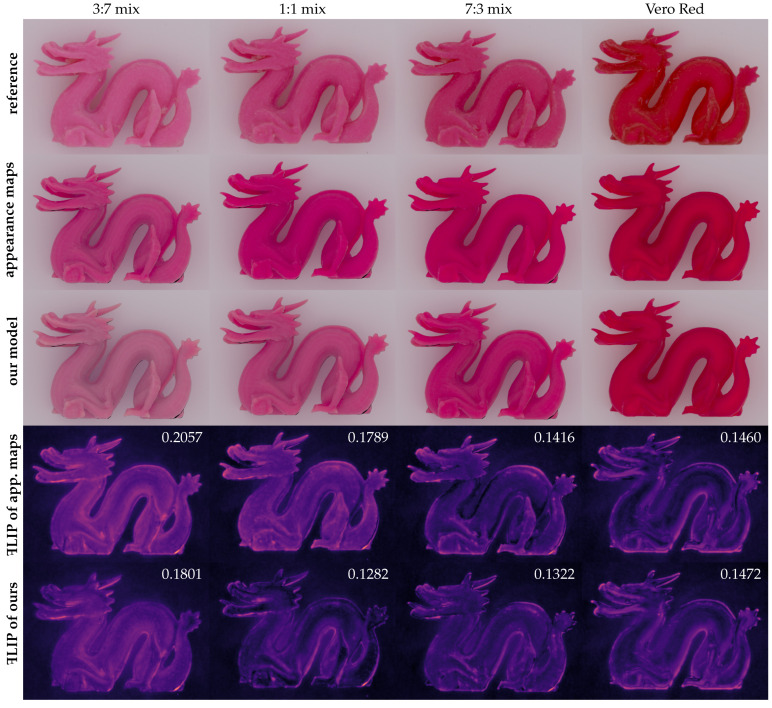
The dragon objects from Figure 3 were PolyJet 3D printed using different mixtures of the materials Vero Red and Vero Pure White. The references are diffusely illuminated multispectral images converted to sRGB. We show images rendered based on our radiometric validation procedure (Section 5) using optical properties estimated with appearance maps [4] and our model (Section 4), and we include the FLIP error maps [8] and average FLIP errors of the rendered images (an average of less than 0.15 seems a reasonably good prediction of the appearance of the printed object).

Figure 10 provides a photo-render comparison of RGB images for the case of different material mixtures. Note that our photo-render alignment and the FLIP error metric enable us to compute one number (average FLIP error) between 0 and 1 to indicate the deviation in the appearance of the rendered digital twin from the appearance of the 3D printed object. In addition, our method enables us to compute the root mean squared error (RMSE) per wavelength (examples included in Figure 1).

## 8. Discussion

**Figure 11:**
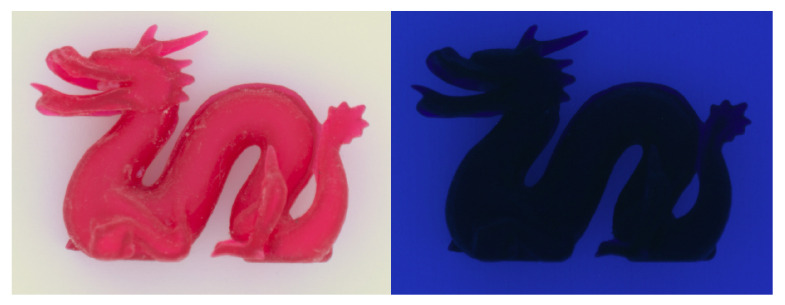
sRGB reconstruction of an unfiltered multispectral Videometer image (**left**) and fluorescence activated by a source with a wavelength of 365 nm (**right**).

Our multispectral images provide additional information about fluorescence in the studied materials. In the outset, our instrument for capturing reference images has narrow-band sources and a monochromatic camera. We first assumed no fluorescence and expected the image captured when using a source of a particular waveband to only include light reflected at this waveband. This would result in purple shadows with too much blue light (Figure 11, left). Applying long pass filters that mask out the waveband of the source, we could observe the amount of fluorescent light (Figure 11, right) scattered to other wavelengths. Subtracting the fluorescent light from the unfiltered images removed the error of too much blue light. However, this also means that our images do not include the fluorescent light that would be shifted to other wavebands, and this is also not captured in our renderings. We leave further investigations on fluorescence for future work.

**Figure 12:**
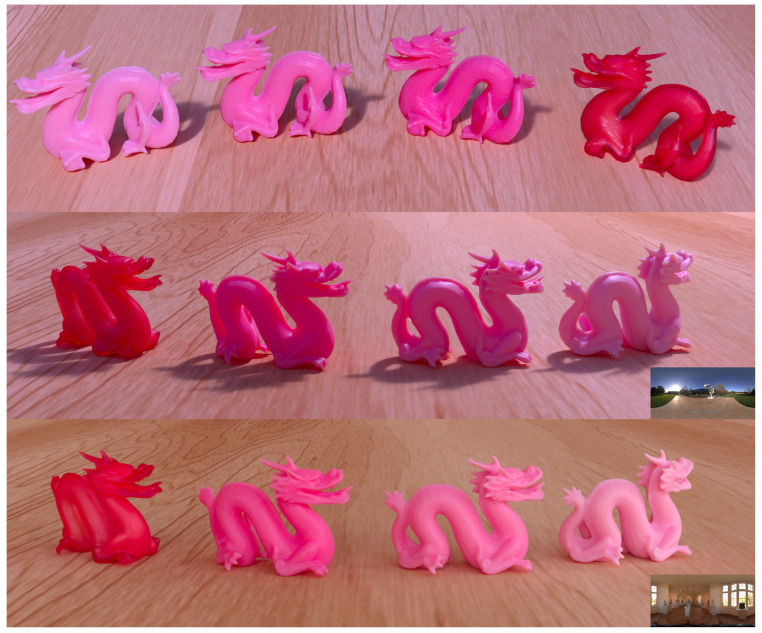
Predicted appearance of PolyJet 3D printed objects with different material mixtures of varying translucency (blending Vero Red and Vero Pure White). For the image at the **top**, the scene was set up to be qualitatively comparable to the photo in Figure 3. In the **middle**, the same scene is seen from a different view. At the **bottom**, we used a different lighting environment. The lighting environments are shown as small inserts in the lower right corner (the **top** and **middle** used the same).

For the material mixtures, we used a linear approximation of the absorption and scattering coefficients. Our validation procedure is useful for testing the validity of such an approach (as seen in Figure 10). The alternative to linear approximation between pure print materials would be to run the full estimation of optical properties for different mixtures. Figure 12 showcases different renderings based on our estimated optical properties. The rendering time depends neither on the physical size of the object nor on the 3D printer’s resolution. It depends on the optical thickness of the material, the resolution of the rendered image, and the lighting environment in the digital scene. For a lighting environment with a fairly uniform distribution of incident illumination, a converged image is obtained in seconds. For a lighting environment with a very localized light source (like the sun), several minutes are required to obtain a converged image. Rendering time can be reduced significantly if denoising is employed [45].

Figure 12 includes a rendered image set up to be qualitatively comparable to the photo in Figure 3. However, one should keep in mind that in the rendering, the lighting environment and the wood underneath the printed objects are different from the surroundings in the photo. Nevertheless, we can use such a rendering (and similar renderings using other captured lighting environments) to qualitatively assess how well the estimated optical properties and selected rendering techniques predict the appearance of our printed objects. However, we would like to emphasize that our validation procedure as seen in Figure 1 and Figure 10 provides an option for the quantitative assessment of the ability of the estimated optical properties to describe the appearance of the printed object. This quantitative assessment is a strong indicator of whether we can use the digital twin with the estimated optical properties to render photorealistic images of the object in an arbitrary digital lighting environment. It is also an important tool for identifying wavelengths where the estimated optical properties have lower accuracy.

In our multispectral images, it was mostly the paper that exhibited fluorescence, so the lack of fluorescence simulation in the rendering likely has a small effect on the rendered result, but this could be different for different materials. In general, the iterative application of our full pipeline (estimation and validation) could be used to investigate the effect on scattering and absorption properties that results from the use of different chemical components in the mixing of 3D printing materials.

In practice, 3D printed objects are oftentimes subject to post-processing like sandblasting or polishing. Such modifications affect surface reflectance. While our work assumes surfaces with a spatially uniform roughness, future studies could better describe surfaces created using different 3D printing techniques or post-processed surfaces. Our model for estimating optical properties currently assumes a uniform high roughness. This would be suitable for sandblasted objects as well. The model would need modification to work for objects polished to have a very smooth surface. The rendering technique used for radiometric validation would need to adapt to model different types of surface roughness. Some existing works use a spatially varying roughness to model different types of surfaces [40].

## 9. Conclusions

For 3D printing processes that produce surfaces with a reasonably uniform surface roughness, we provided a practical method for acquiring spectral absorption and scattering properties of translucent materials. Our model is practical due to being analytic and for easily printed thin slabs, which eases the fitting of the model to reflectance and transmittance measurements acquired with a conventional spectrophotometer. We proposed a procedure for aligning the digital twin and camera with a multispectral photo of a diffusely illuminated physically printed object. This enables the use of pixel-to-pixel photo-render comparison of individual spectral images and reconstructed RGB images. In turn, this enables the use of quantitative full-reference-image quality metrics such as the RMSE and FLIP error for assessing the appearance prediction capabilities of the digital twin extended with estimated optical properties. To this extent, we met our objectives. Our method for estimating optical properties is not perfect, but the point of our radiometric validation method was also to identify where estimated optical properties are of lower accuracy. We propose that the photo-render alignment can be used to further refine the spectral optical properties and perhaps also for estimating scattering properties describing the directional distribution of the scattered light. Hopefully, our work can inspire others to more often conduct a quantitative validation of appearance prediction in 3D printing through photo-render comparisons. We found that quantitative comparisons provide a different and important level of additional information on top of the conventional side-by-side juxtaposition of renderings and photographs for visual inspection.

## Data Availability

The raw data supporting the conclusions of this article will be made available by the authors upon request.
